# Supplementary material for: Structure insight into FtsZ function maintaining under acid stress of Streptococcus mutans
Source: Int J Oral Sci. 2026 Jan 4;18:3. doi: 10.1038/s41368-025-00400-9 (PMC12764573; doi:10.1038/s41368-025-00400-9)
Supplement: Supplementary file 1 — Supporting Information [file 41368_2025_400_MOESM1_ESM.pdf]

Supporting Information for

Structure Insight into FtsZ Function Maintaining Under Acid Stress of *Streptococcus mutans*

Yuxing Chen[1*], Yongliang Li[1*], Jiahao Niu[2], Liuchang Yang[1], Yaqi Chi[1], Xue Cai[1], Fengjiao Xin[3], Jie Zhang[4,5], Xianyang Fang[5], Yi Qin Gao[6,7,8], Manas Mondal[6#], Xiaoyan Wang[1#]

* Yuxing Chen and Yongliang Li contributed equally to this work.

# Address correspondence to Manas Mondal, manas@szbl.ac.cn, or Xiaoyan Wang, wangxiaoyan@pkuss.bjmu.edu.cn.

This PDF file includes:

Supporting text
Supplementary Figures S1 to S16
Supplementary Tables S1 to S5

Supporting Information Text

Molecular Dynamics simulation studies on interactions at the binding interface of SmFtsZ in acidic pHs environments

Binding interface & stability of secondary structural elements of SmFtsZ in dimeric complex. From our all-atom MD simulation studies SmFtsZ chains are found to maintain the dimeric assembly at different pHs through interactions among the interface residues as depicted in the residue contact maps (Fig. S13). We found that residues 134-150 and 165-190 of chain-A, which includes H5 and H6 helices mainly form the interface contacts with the residues 200-222 and 258-300 of chain-B that contains H8 and H10 helices, S8 and S9 sheets and T7 loop in SmFtsZ-dimer$_{long}$ case (Fig. S5, S13a-c). On the other side in SmFtsZ-dimer$_{late}$ complex, residues 40-96 contain H2 and H3 helices, S2 and S3 sheets and T3 loop in the N-terminal domain of chain-A form the interface contacts with the resides 224-314 include H9 and H10 helices in the C-terminal domain of chain-B (Fig. S5, S13d-f). Variation of secondary structural elements of SmFtsZ chains in SmFtsZ-dimer$_{long}$ and SmFtsZ-dimer$_{late}$ complexes over the simulation time (Fig. S14, S15) does not show any major change in folded conformation of protein chains at acidic pH environment. However, N-terminal H1 (21-34) and H2 (48-53) helices in GTP-binding pocket of chain-A, H8 (212-219) helix of chain-B, and H5 (142-158) and H6 (167-173) helices of chain-A and chain-B show differential stability at pH 5.0, 6.0 and 7.0 in SmFtsZ-dimer$_{long}$ complex (Fig. S14). Similarly, pH dependent different stability of H5, H6 and H7 (180-203) helices of both the protein chains are found in case of SmFtsZ-dimer$_{late}$ (Fig. S15).

Interface area and binding interactions in SmFtsZ dimeric complex. We analyzed the total buried surface area (BSA) and BSA of the individual protein chains, which measures the size of the interface in the dimeric complexes in different pH environment. In case of SmFtsZ-dimer$_{long}$, average total BSA is comparatively higher with greater contribution from chain-A at pH 6 (Fig. S9a). Average non-bonded interaction energy (Eint) is also comparatively high at pH 6 (Fig. S9b), where electrostatic interactions mainly favor the dimeric association. At the same time binding interaction of GTP with chain-A is more favored at pH 6 (Fig. S9c). As compared to the physiological pH, Eint between the protein chains in SmFtsZ-dimer$_{late}$ complex is higher at acidic pHs (Fig. S9d), and total BSA and BSA of the individual protein chains attain higher value at pH 5 (Fig. S9e). The average number of potential hydrogen bonds (NH) between the protein chains show comparatively higher value at pH 6 for SmFtsZ-dimer$_{long}$ and at pH 5 and 6 for SmFtsZ-dimer$_{late}$ (Fig. S9f-g).

At different pH environment interactions among the key interface residues in SmFtsZ-dimer$_{long}$ complex. We studied the key interactions among the interface residues in SmFtsZ-dimer$_{long}$ complex simulated at different pHs. Fig. S11a-c show the interface residues in SmFtsZ-dimer$_{long}$, which form favorable hydrogen bonds between the protein chains and stabilize the GTP in the binding pocket of chain-A at pH 5.0, 6.0 and 7.0. Arg144 of chain-A forms salt-bridge with Asp214 of chain-B both at pH 7.0 and 6.0. At pH 7.0 backbone atoms of F139 and K143 of chain-A form favorable hydrogen bonds with the L295 and N292 of chain-B, respectively (Fig. S11a). Besides, Asn285, Leu180 and Phe139 of chain-A form potential hydrogen bonded interactions with Lys150, Met271 and Ser280 of chain-B, respectively at pH-

6 (Fig. S11b). Ile293 of chain-B also forms hydrogen bonded interactions with Ser142 and Lys143 of chain-A at pH 5.0 and 6.0. Salt-bridge interaction of Arg144-Asp214 is disrupted at pH 5.0 due to protonation of side chain of Asp214. However, protonation of side chain of Asp211 of chain-B and Glu140 of chain-A assists to form the hydrogen bonded contact with the phosphate group of GTP and helps to stabilize at acidic pHs (Fig. S11b,c).

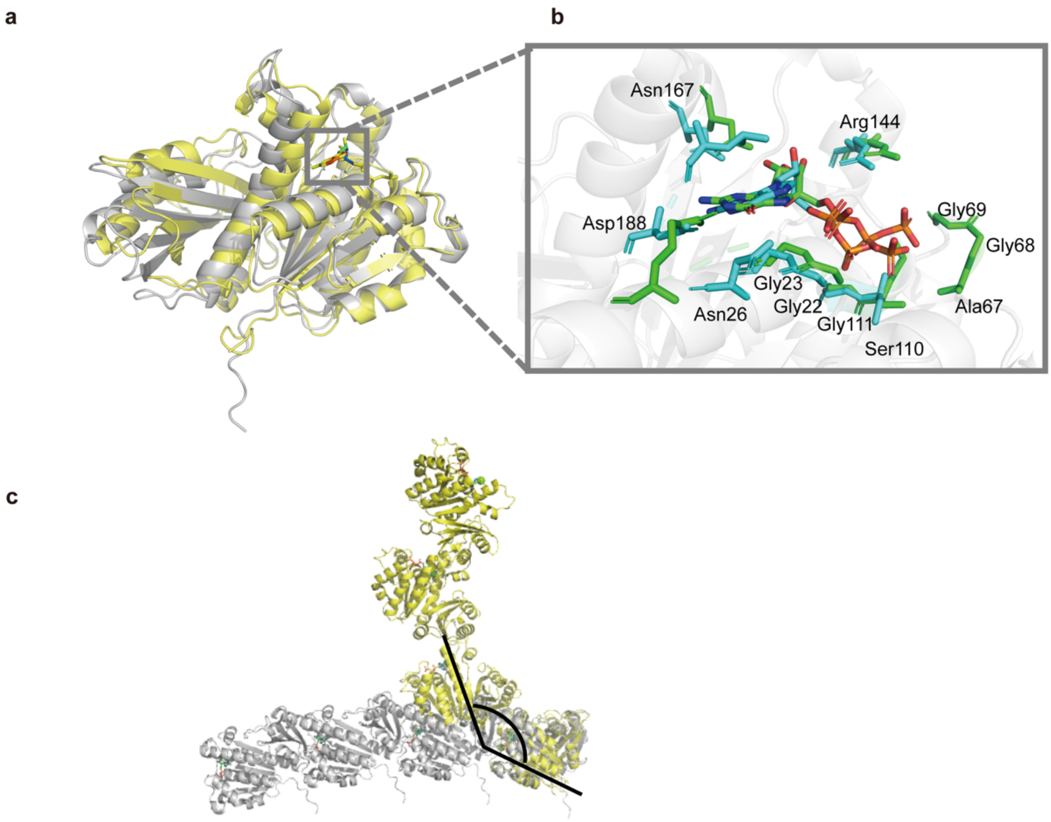

Supplementary Fig. S1. a Superposed monomer of SmFtsZ solved from crystal structure (yellow) and predicted by AlphaFold3 (gray). **b** GTP binding sites were shown. **c** Superposed tetramers of SmFtsZ solved from crystal structure (yellow) and predicted by AlphaFold3 (gray).

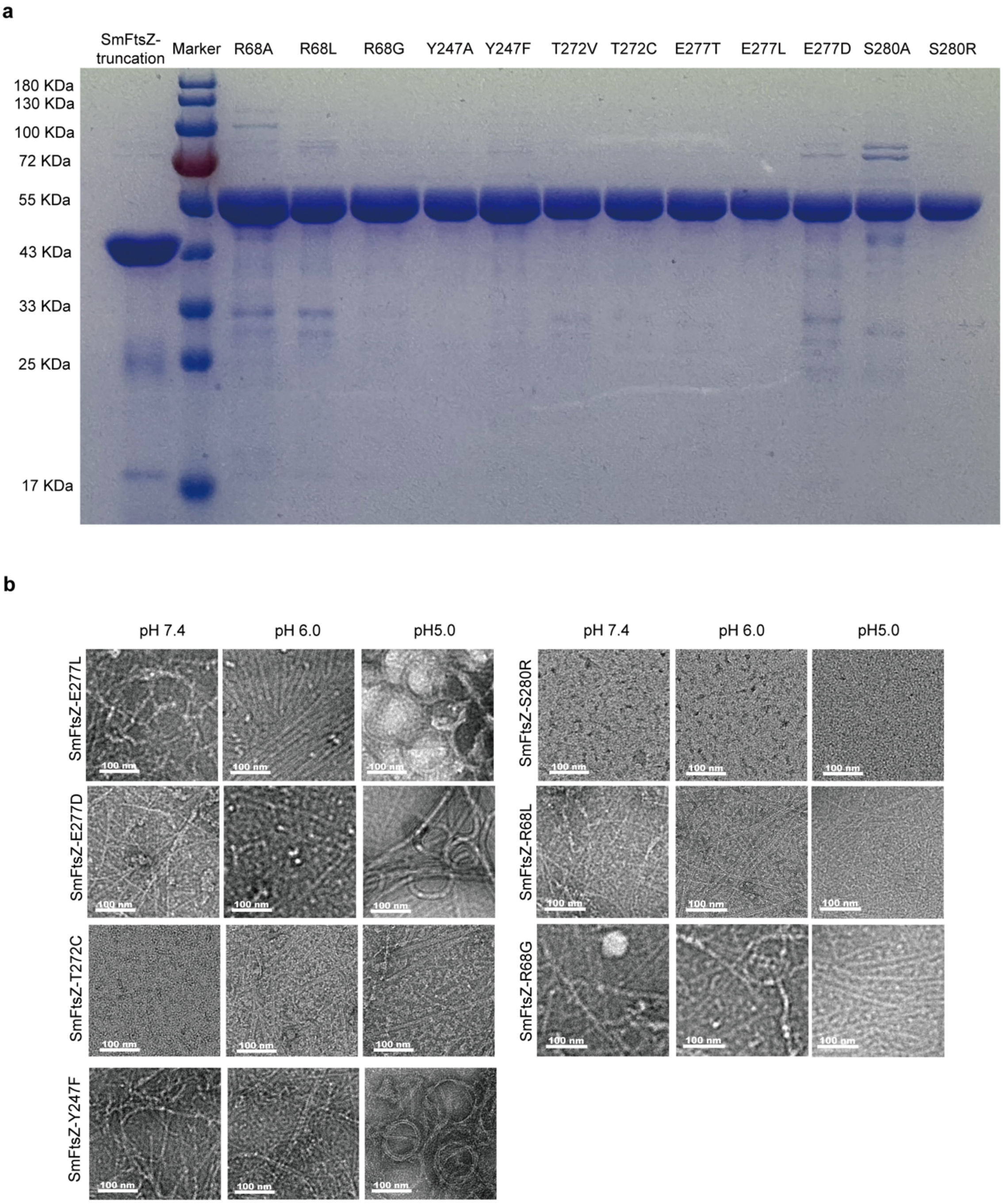

Supplementary Fig. S2. a Sodium dodecyl sulfate-polyacrylamide gel electrophoresis analysis of SmFtsZ-truncation and SmFtsZ mutation. Lane M: protein marker. **b** The polymerization of SmFtsZ mutation at different pH values.

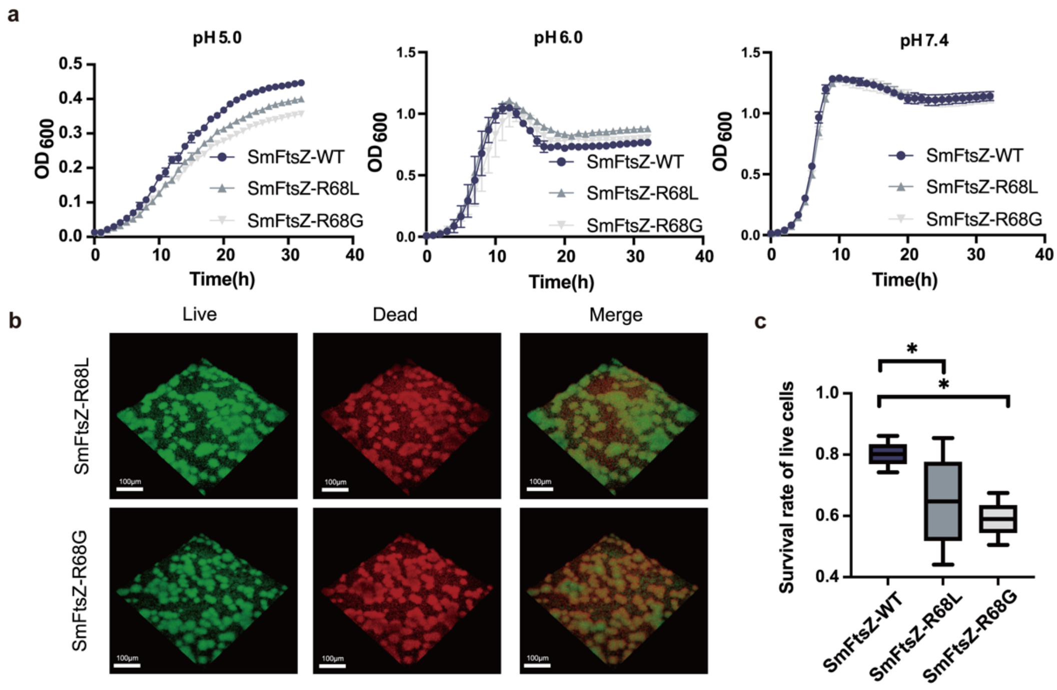

Supplementary Fig. S3. Effects of an acidic environment on *S. mutans* UA159 strain and SmFtsZ-R68L/G strains. **a** Growth curves of *S. mutans* UA159 strain and SmFtsZ-R68L/G strains under different pH levels. Data were obtained from three independent experiments. **b** Representative CLSM images of 96 h biofilm of *S. mutans* UA159 strain and SmFtsZ-R68 L/G strains. Lived cells (green) were stained with SYTO 9 stain, and dead cells (red) were stained with propidium iodide. Images were examined at 25× objective magnification. Scale bar: 100 μm. **c** Ratio of live cells of biofilm analysis using Leica imaging software. Data represent the means of three independent experiments. '*' indicates $p < 0.05$ (compared with SmFtsZ-WT respectively with t-test).

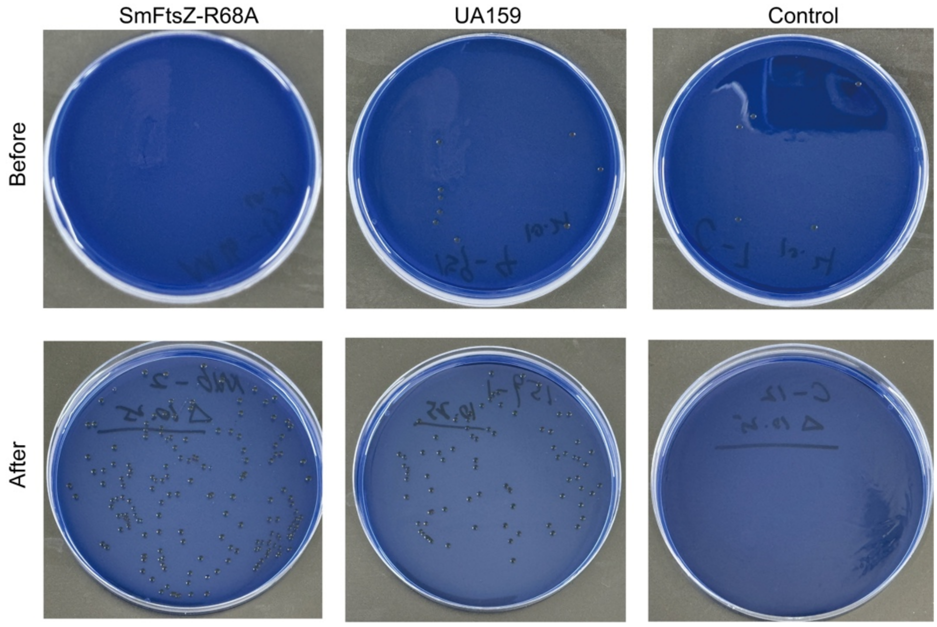

Supplementary Fig. S4. Colonies of *S. mutans* UA159 and SmFtsZ-R68A strain were successfully detected after oral inoculation. Oral samples are collected from rats' mouth using a small swab, including saliva and bacteria colonized on the tooth surface. These samples were spread onto mitis-salivarius-bacitracin agar (MSA) plates. "Control" indicates negative control, we use sterilized PSB instead of bacteria for oral inoculate in animal model.

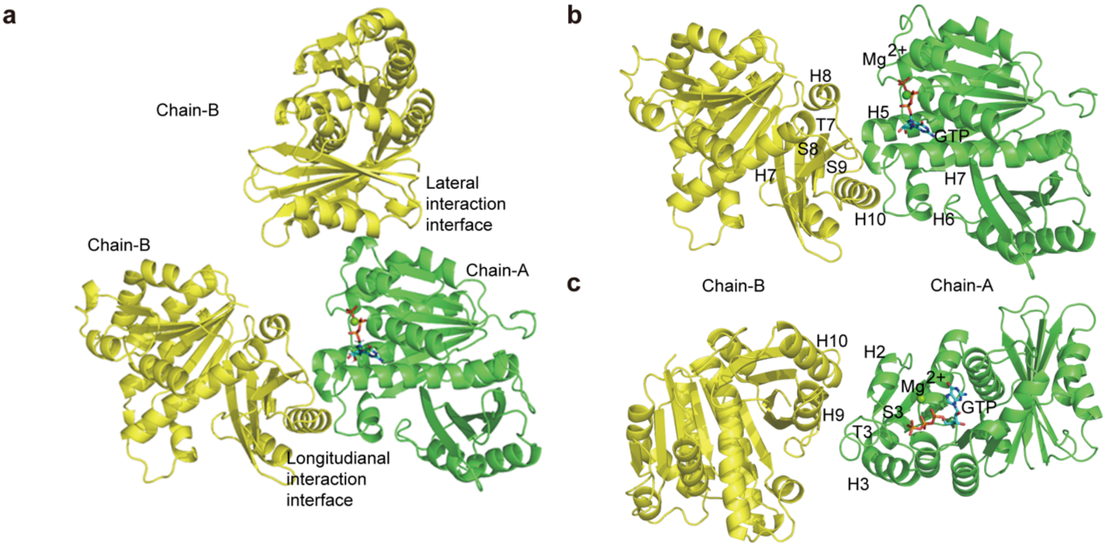

Supplementary Fig. S5. Model structure of SmFtsZ dimer with longitudinal SmFtsZ-dimer$_{long}$ and lateral SmFtsZ-dimer$_{late}$ interaction interfaces(**a**), and structure of (**b**) SmFtsZ-dimer$_{long}$ and (**c**) SmFtsZ-dimer$_{late}$ for molecular dynamics simulations studies.

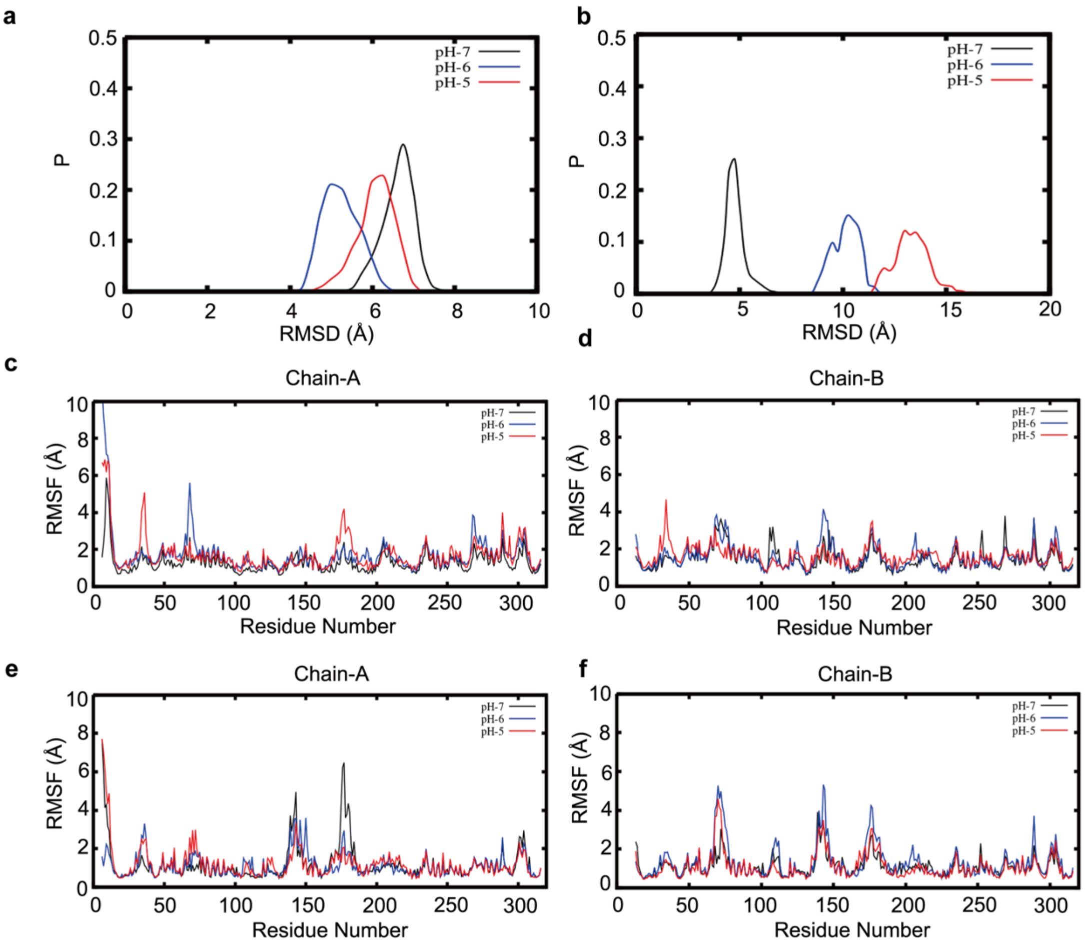

Supplementary Fig. S6. Distribution of the root mean square deviations (RMSD) of the (**a**) SmFtsZ-dimer$_{long}$ and (**b**) SmFtsZ-dimer$_{late}$ structures with respect to the initial structure, and root mean square fluctuations of the residues in the individual protein chains in (**c-d**) SmFtsZ-dimer$_{long}$ and (**e-f**) SmFtsZ-dimer$_{late}$ complexes over the equilibrated MD trajectories at different pHs.

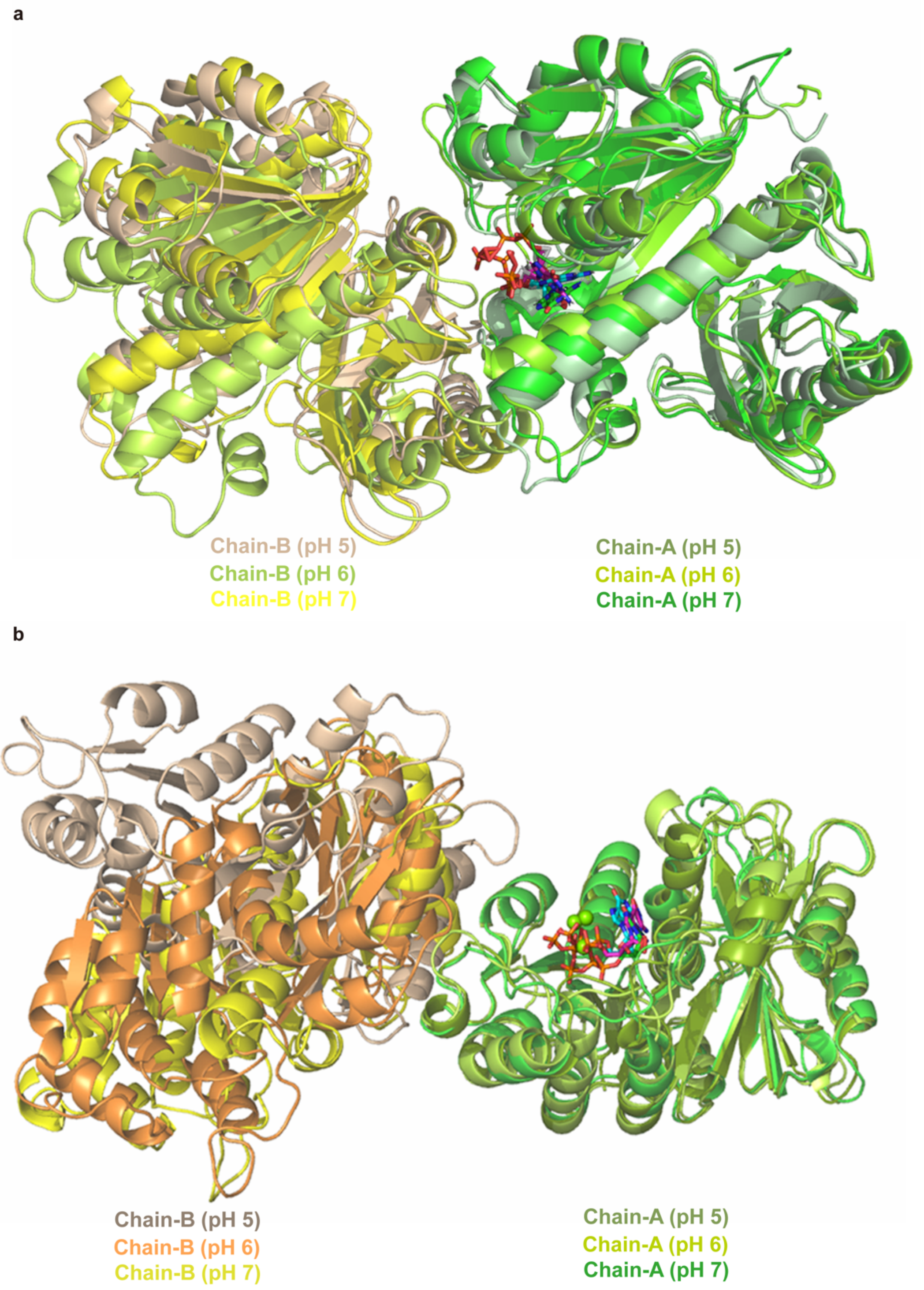

Supplementary Fig. S7. Superposed simulated average structure of (**a**) SmFtsZ-dimer$_{long}$ and (**b**) SmFtsZ-dimer$_{late}$ at pH 7.0, 6.0 and 5.0.

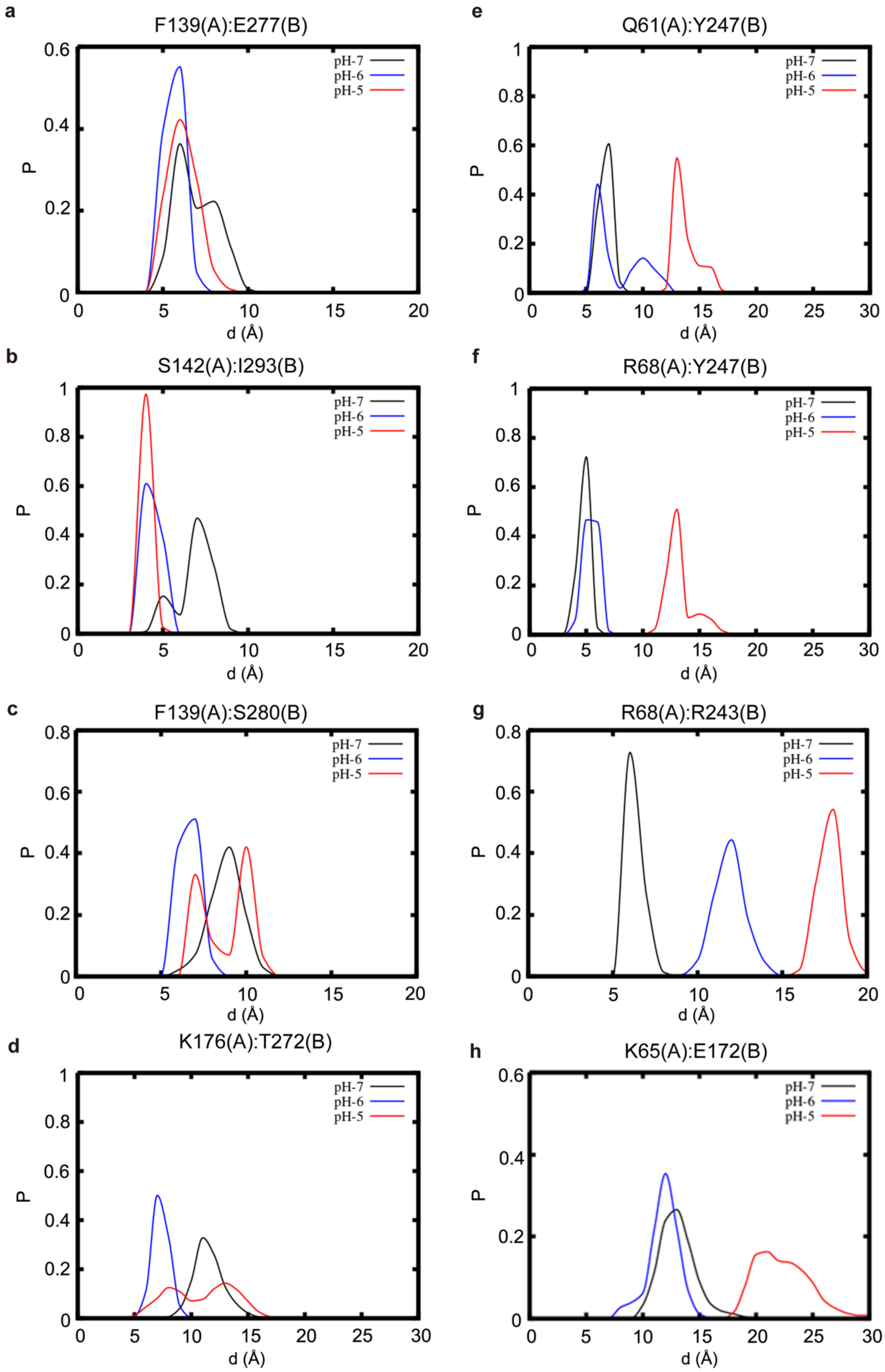
a
F139(A):E277(B)
b
S142(A):I293(B)
c
F139(A):S280(B)
d
K176(A):T272(B)
e
Q61(A):Y247(B)
f
R68(A):Y247(B)
g
R68(A):R243(B)
h
K65(A):E172(B)
pH-7
pH-6
pH-5
P
d (Å)

167

Supplementary Fig. S8. Distribution of the distances of representative residues pairs of protein chains in case of (**a-d**) SmFtsZ-dimer$_{long}$ and (**e-h**) SmFtsZ-dimer$_{late}$ at different pHs, which primarily form the contacts in the dimeric interface, as found in the initial crystal structure.

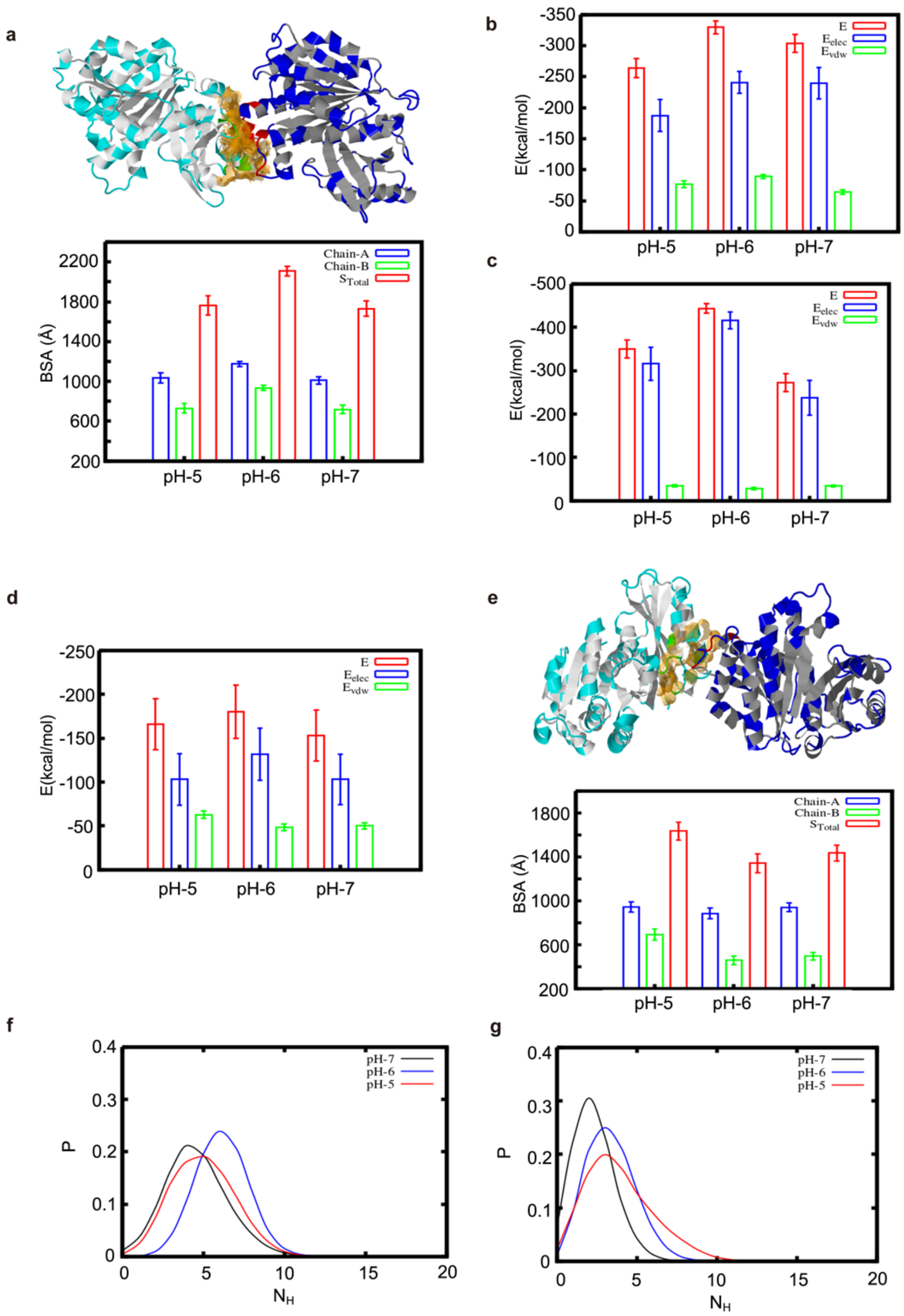

Supplementary Fig. S9. In case of SmFtsZ-dimer$_{long}$ (**a**) total buried surface area (BSA) and BSA of the individual protein chains, and non-bonded interactions energy between the (**b**) protein chains and (**c**) chain-A with GTP at different pHs. **d** Non-bonded interactions energy between the protein chains and (**e**) total BSA and BSA of the individual protein chains in SmFtsZ-dimer$_{late}$ complex at different pHs. Distribution of the number of favorable hydrogen bonds between the protein chains over the equilibrated simulated trajectories in case of (**f**) SmFtsZ-dimer$_{long}$ and (**g**) SmFtsZ-dimer$_{late}$ at different pHs.

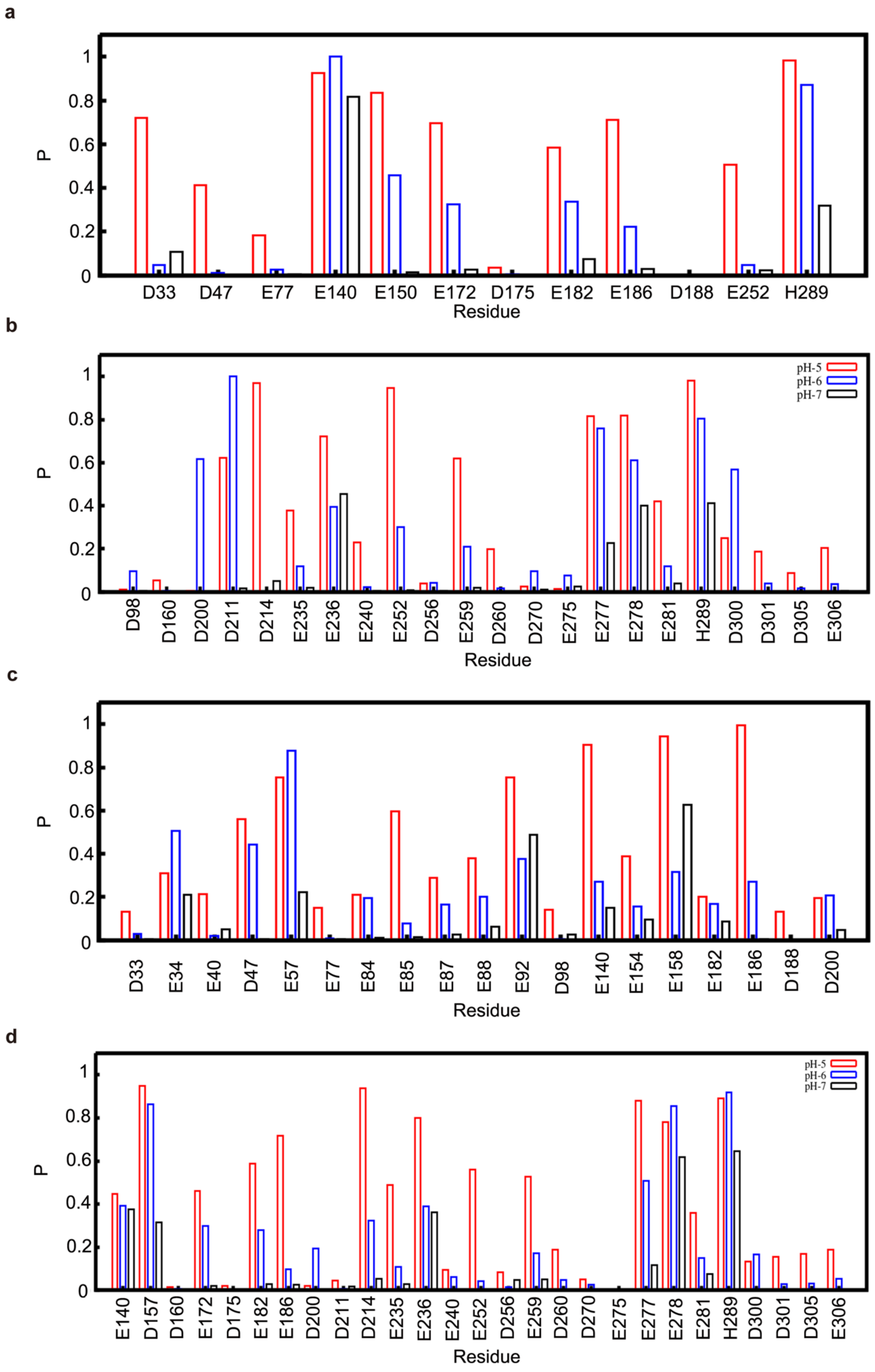
a
P
Residue
D33 D47 E77 E140 E150 E172 D175 E182 E186 D188 E252 H289
b
pH-5
pH-6
pH-7
P
Residue
D98 D160 D200 D211 D214 E235 E236 E240 E252 D256 E259 D260 D270 E275 E277 E278 E281 H289 D300 D301 D305 E306
c
P
Residue
D33 E34 E40 D47 E57 E77 E84 E85 E87 E88 E92 D98 E140 E154 E158 E182 E186 D188 D200
d
pH-5
pH-6
pH-7
P
Residue
E140 D157 D160 E172 D175 E182 E186 D200 D211 D214 E235 E236 E240 E252 D256 E259 D260 D270 E275 E277 E278 E281 H289 D300 D301 D305 E306

Supplementary Fig. S10. Protonation probability of the titrated Asp, Glu and His residues of chain-A and chain-B at pH 7.0 (black), 6.0 (blue) and 5.0 (red) over the equilibrated simulated trajectories in case of (**a-b**) SmFtsZ-dimer$_{long}$ and (**c-d**) SmFtsZ-dimer$_{late}$.

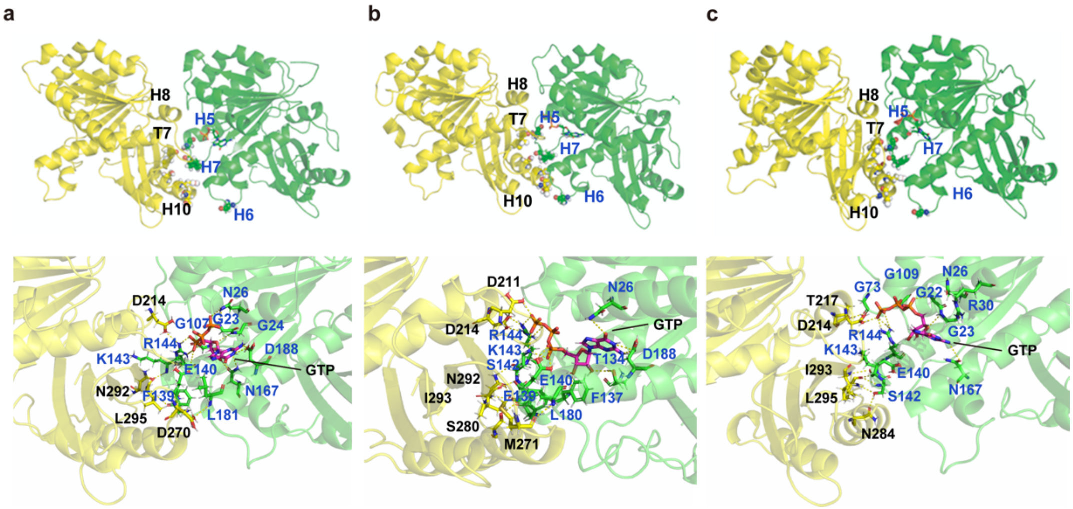

Supplementary Fig. S11. Dimeric binding interface and interface residues, which form potential hydrogen bonds between the protein chains in (**a-c**) SmFtsZ-dimer$_{long}$, complexes at pH 7.0, 6.0 and 5.0, respectively. (chain A and chain B are shown in green and yellow color, respectively)

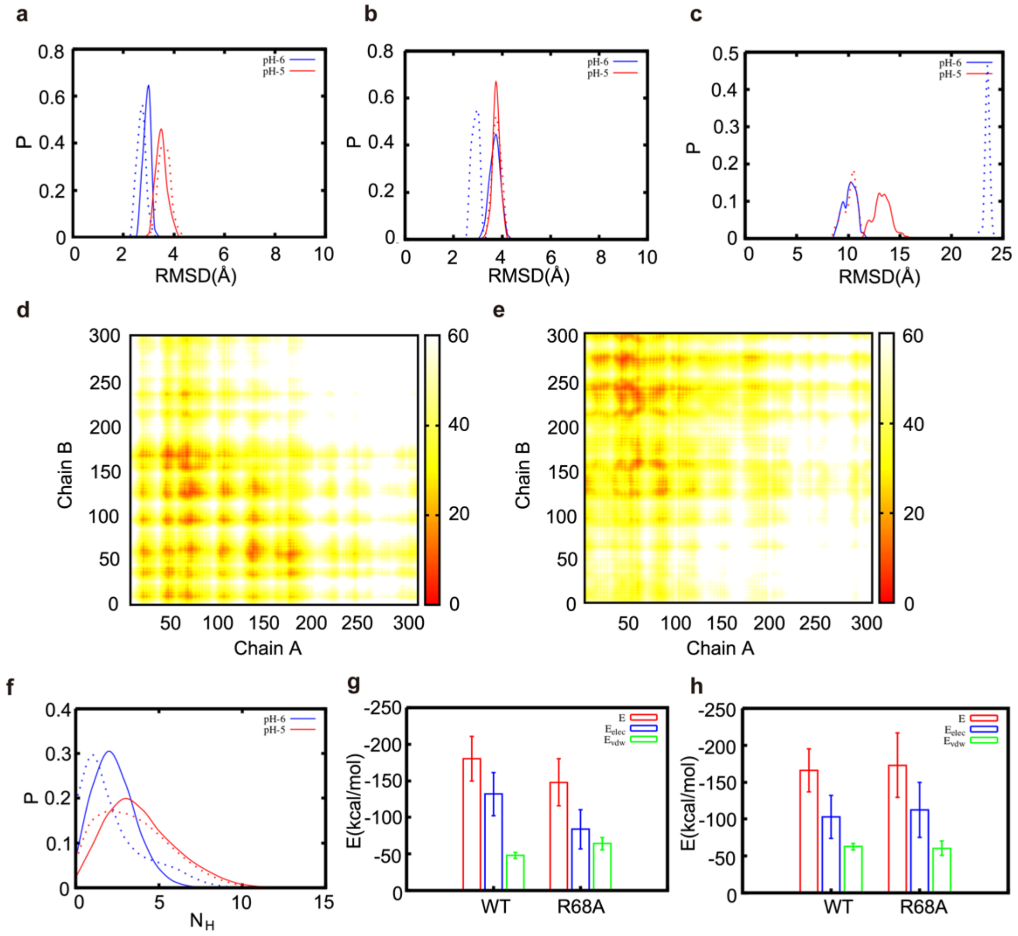

Supplementary Fig. S12. Distribution of the root mean square deviations (RMSD) of the (**a-b**) individual protein chains and (**c**) dimeric lateral complex for wild type (solid line) and R68A mutated (dotted line) case with respect to the initial structure over the equilibrated MD trajectories at pH 5.0 and 6.0. **d**, **e** Residues contacts map between the protein chains in dimeric lateral complex for R68A mutated case over the equilibrated MD trajectories at pH 6.0 and 5.0, respectively. The color bar represents the pair distance between the residues of protein chains. **f** Distribution of the number of favorable hydrogen bonds between the protein chains in lateral dimeric complex over the equilibrated simulated trajectories at pH 6 and 5 for SmFtsZ-WT (solid line) and SmFtsZ-R68A (dotted line) case. **g, h** Non-bonded interaction energy between the protein chains in lateral dimeric complex for SmFtsZ-WT and SmFtsZ-R68A case at pH 6.0 and 5.0, respectively.

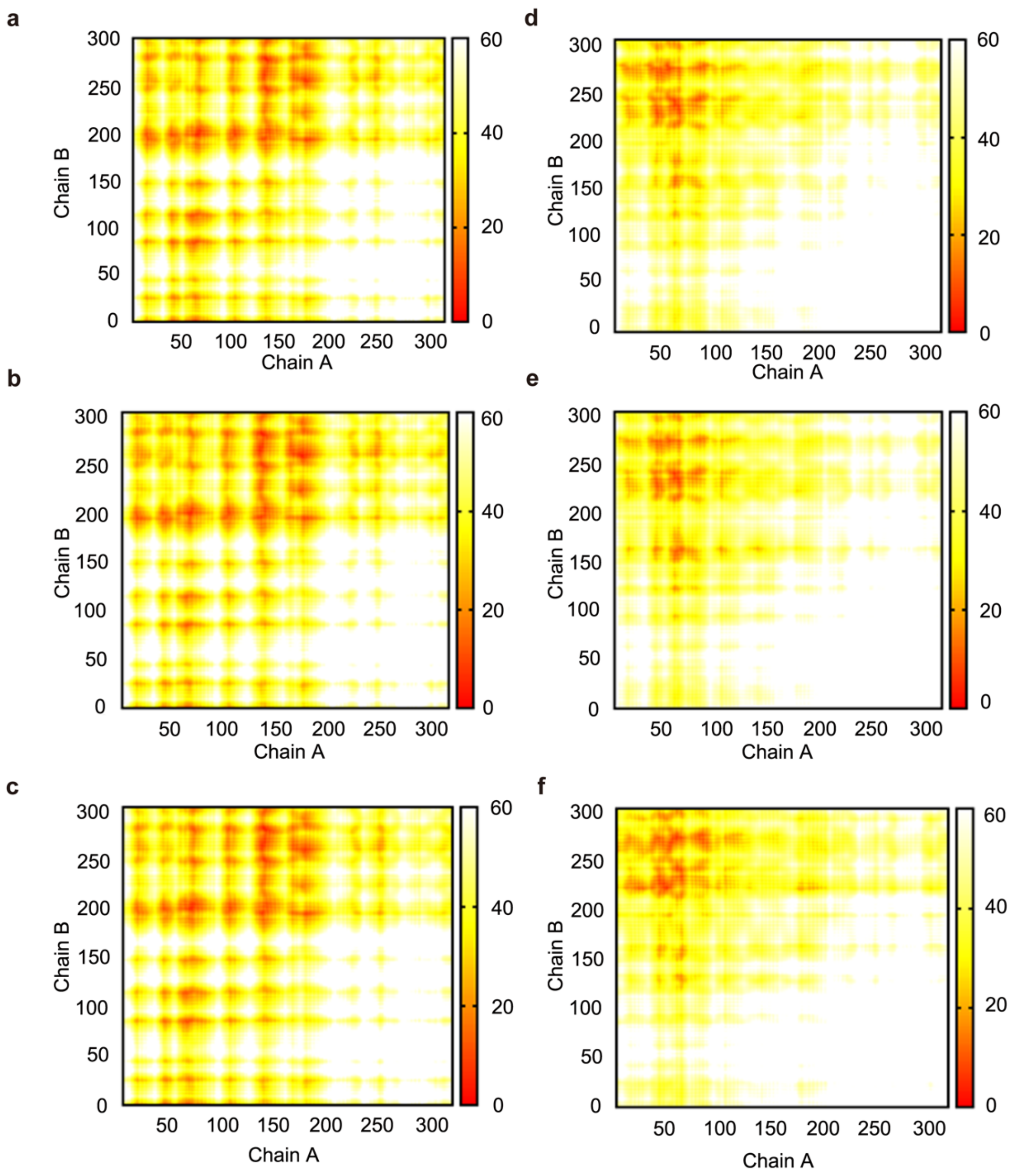

Supplementary Fig. S13. Residues contacts map between the protein chains in (**a-c**) SmFtsZ-dimer$_{long}$ and (**d-f**) SmFtsZ-dimer$_{late}$ complexes over the equilibrated MD trajectories at pH 7.0, 6.0 and 5.0, respectively. The color bar represents the pair distance between the residues of protein chains.

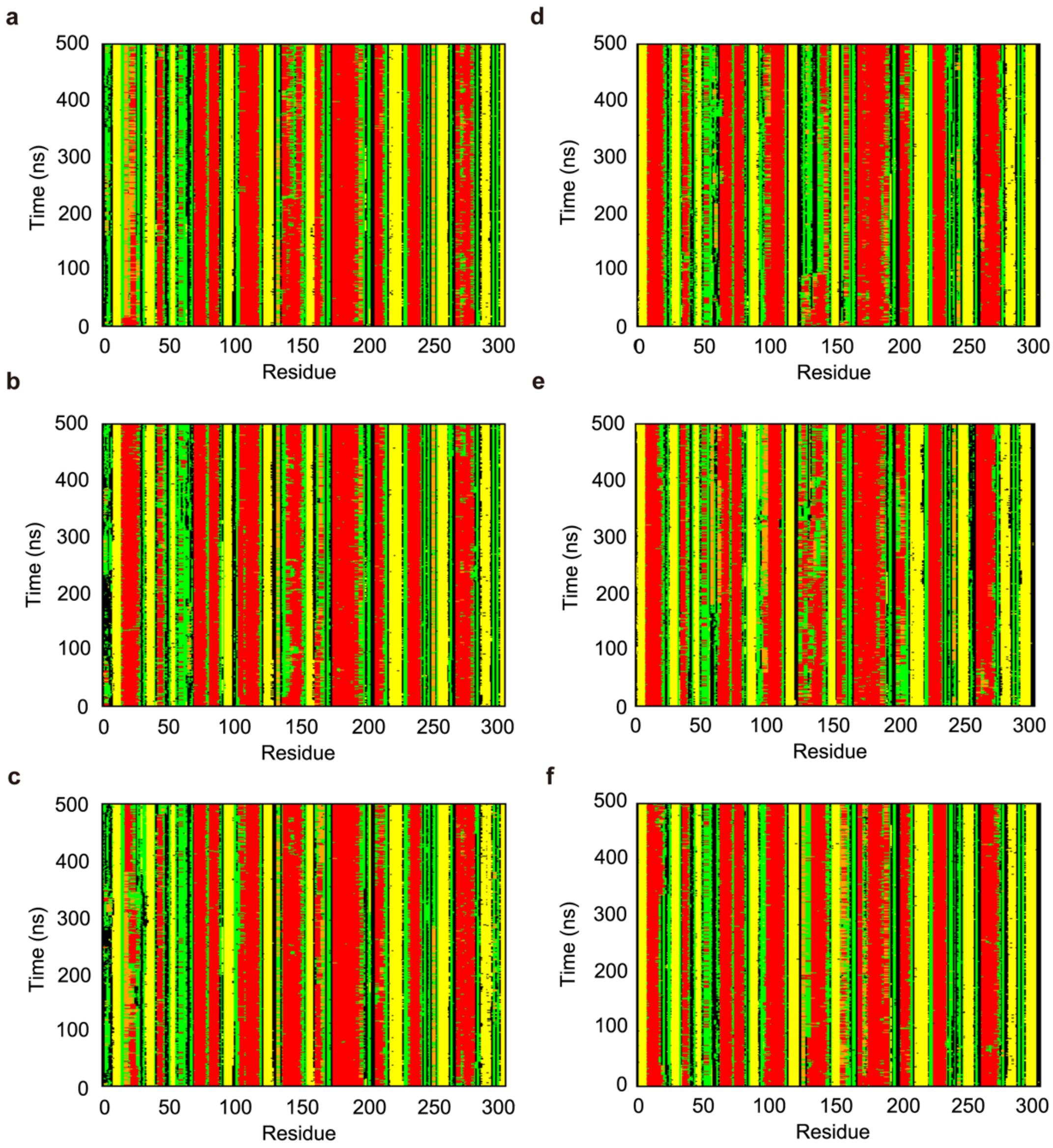

Supplementary Fig. S14. Secondary structural preference of (**a-c**) chain-A and (**d-f**) chain-B at pH 7.0, 6.0 and 5.0, respectively over the simulation time in SmFtsZ-dimer$_{long}$ complex. (Secondary structural components are represented as α-helix: brown, 3-10 Helix/pi-Helix: orange, β-sheet: yellow, Turn/Bend: green).

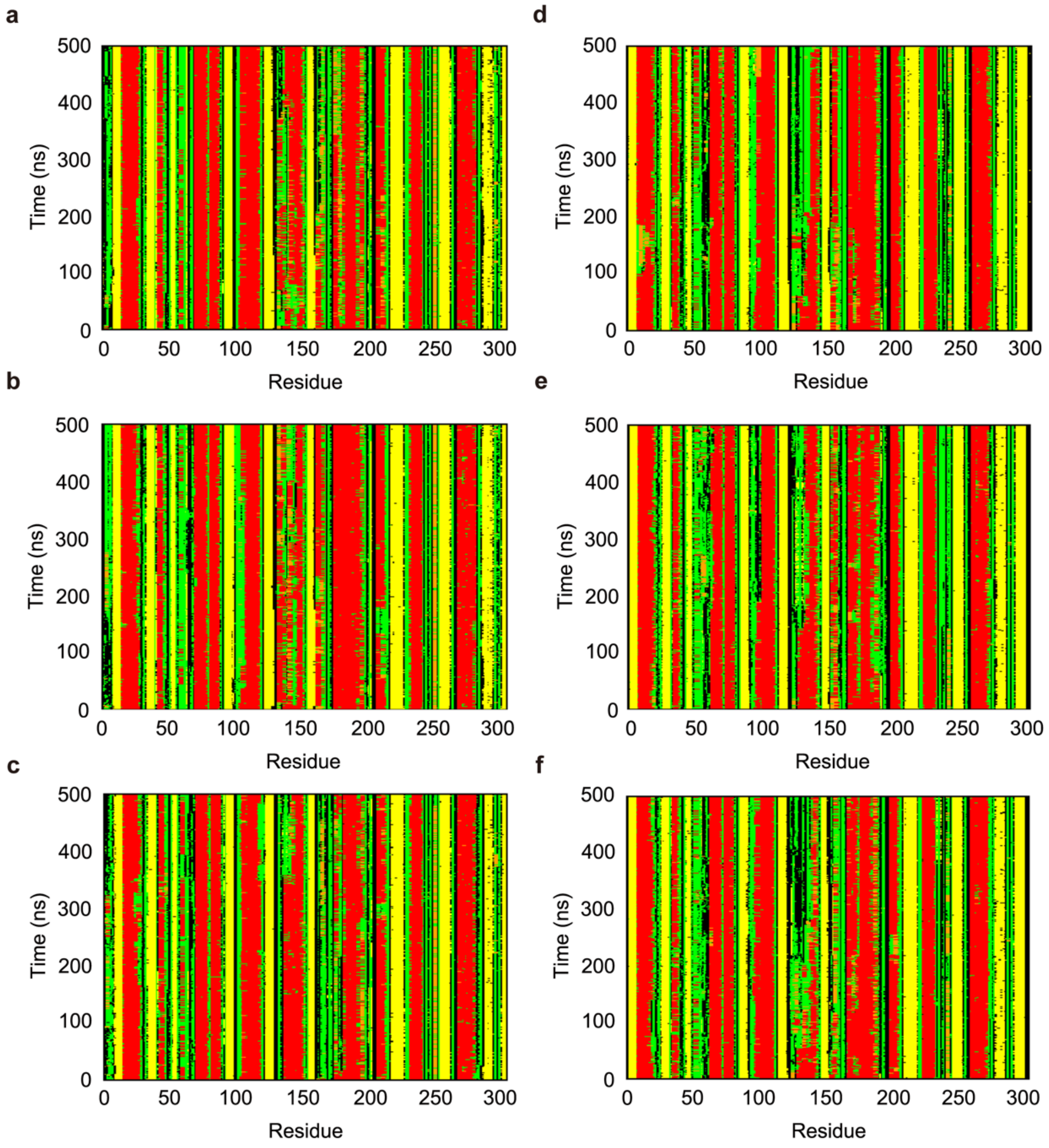

Supplementary Fig. S15. Secondary structural preference of (**a-c**) chain-A and (**d-f**) chain-B at pH 7.0, 6.0 and 5.0, respectively over the simulation time in SmFtsZ-dimer$_{late}$ complex. (Secondary structural components are represented as α-helix: brown, 3-10 Helix/pi-Helix: orange, β-sheet: yellow, Turn/Bend: green).

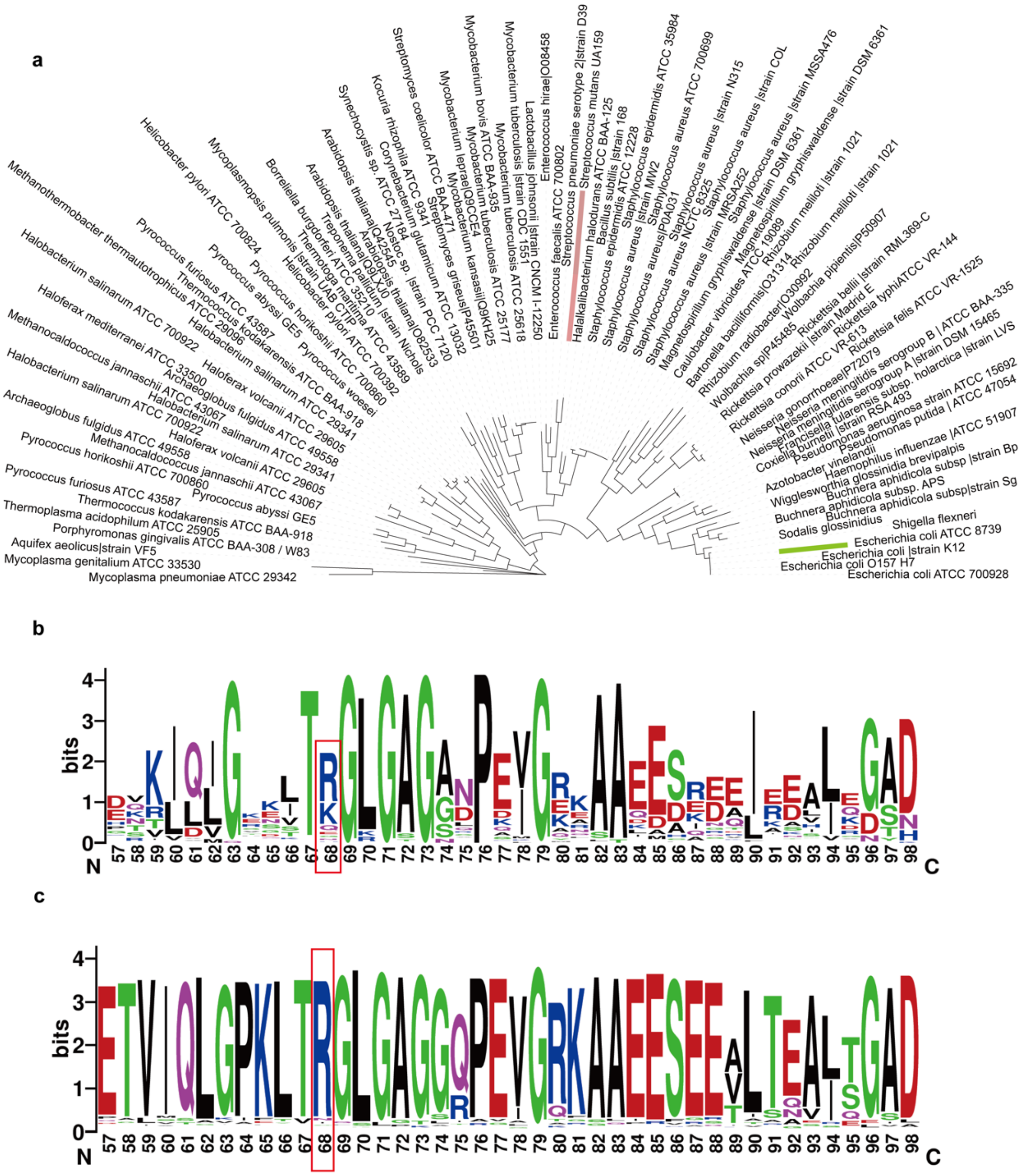

Supplementary Fig. S16. Sequence alignment of FtsZ homologs. **a** Phylogenetic analysis of FtsZ sequences. A Maximum Likelihood tree was constructed using the MUSCLE in MEGA, illustrating evolutionary relationships among species. **b** Partial sequence alignment of FtsZ homologs focusing on the 68th amino acid position. Sequence logos were generated using WebLogo 3.6, where the size of each amino acid letter reflects its frequency at each aligned position across species. **c** Subgroup-specific alignment of FtsZ sequences from Streptococcus (n=506). Sequence logos (WebLogo 3.6) depict amino acid frequency at each position, with the 68th residue highlighted for conservation analysis.

Supplementary Table S1. Data collection and refinement statistics

**Data collection**	
Space Group	$P4_32_12$
Cell dimensions	
a, b, c (Å)	173.12 173.12 172.28
α, β, γ, (°)	90 90 90
Resolution (Å)	34.52- 3.49 (3.62- 3.49)
R_{merge} (%)	0.173 (1.075)
I / σI	10.67 (1.18)
Completeness (%)	90.08 (51.84)
Redundancy	9.6 (9.1)
**Refinement**	
No. reflections	30527 (1723)
R_{work} / R_{free} (%)	25.75/28.74
No. atoms	
Protein	8772
Ligands	4
Water	10
Average B-factor	
Protein	55.60
Ligands	18.61
Water	18.61
R.m.s. deviations	
Bond lengths (Å)	
Bond angles (°)	0.003
Ramachandran plot statistics (%)	0.59
Most favoured	95.23
Allowed	4.77
Disallowed	0.0

*Values in parentheses are for highest-resolution shell.

Values in parentheses are for the highest resolution shell. $Rmerge=\Sigma h \Sigma i|Ih,i-Ih|/\Sigma h \Sigma i Ih,i$, where Ih is the mean intensity of the i observations of symmetry related reflections of h. $R=\Sigma|Fobs-Fcalc|/\Sigma Fobs$, where $Fcalc$ is the calculated protein structure factor from the atomic model (Rfree was calculated with 5% of the reflections selected randomly).

Supplementary Table S2. SAXS experimental data statistics table of different samples

	pH	Concentrati on mg/mL	Rg(Å) (Guinier)	I0 (Guinier)	Rg(Å) (Gnom)	I0 (Gnom)	Dmax	molecular weight（kDa）
SmFtsZ-WT	7.4	4	65.5	421	65.8	412	220	218.4
SmFtsZ-R68A	7.4	4	53.2	237	54.7	235	188	166.6
SmFtsZ-WT	6.0	4	135.9	1240	137.4	1157	500	794
SmFtsZ-R68A	6.0	4	93.3	898	96.5	885	300	489

Supplementary Table S3. Gene of acid tolerance expression analysis from RNA-seq results of UA159 strain and SmFtsZ-R68A strain when these strains were cultured in an acidic environment.

gene_id	UA159	SmFtsZ-R68A	FoldChange	log2FoldChange	*p*-value	*q*-value	Regulation	description
SMU_RS01330	58.1076358	27.1278474	0.46641494	-1.1003141	0.0004182	0.00069927	Down	putrescine carbamoyltransferase
SMU_RS01335	27.57447371	18.7230753	0.67232089	-0.5727781	0.21405343	0.24998566	Down	APC family permease
SMU_RS01340	56.60291529	27.1317069	0.47899667	-1.0619125	0.00080353	0.00130439	Down	agmatine deiminase
SMU_RS01345	30.7145229	15.4371421	0.50398433	-0.9885492	0.02054605	0.02811378	Down	carbamate kinase
SMU_RS01325	111.7660616	43.6881021	0.39006324	-1.3582201	1.53E-09	3.87E-09	Down	helix-turn-helix transcriptional regulator
SMU_RS06930	4962.009941	6274.28012	1.26423154	0.33826072	4.15E-06	8.20E-06	Up	MULTISPECIES: F0F1 ATP synthase subunit epsilon
SMU_RS06935	19943.63721	25030.0854	1.25498528	0.32767044	1.54E-05	2.87E-05	Up	F0F1 ATP synthase subunit beta
SMU_RS06940	5918.904059	12832.4122	2.16760824	1.11610403	3.80E-27	2.61E-26	Up	F0F1 ATP synthase subunit gamma

SMU_RS06945	13448.85398	18230.2086	1.35543244	0.4387532	2.54E-05	4.68E-05	Up	F0F1 ATP synthase subunit alpha
SMU_RS06950	2599.872409	4275.15872	1.64394749	0.71716422	7.21E-13	2.26E-12	Up	F0F1 ATP synthase subunit delta
SMU_RS06955	2442.446433	4712.63715	1.92878035	0.94768886	2.11E-16	8.27E-16	Up	F0F1 ATP synthase subunit B
SMU_RS06960	5208.440856	6573.15995	1.26182493	0.33551176	0.00536017	0.00795249	Up	F0F1 ATP synthase subunit A
SMU_RS06965	3536.555905	1756.08159	0.49644955	-1.010281	4.60E-09	1.14E-08	Down	MULTISPECIES: F0F1 ATP synthase subunit C
SMU_RS07895	2401.540682	3457.36685	1.43903668	0.52510337	1.73E-06	3.53E-06	Up	3-hydroxyacyl-ACP dehydratase FabZ
SMU_RS07910	2346.063533	4135.33259	1.76216612	0.81734994	8.95E-17	3.57E-16	Up	3-oxoacyl-[acyl-carrier-protein]
SMU_RS07920	3638.651149	4108.25748	1.12892343	0.17494764	0.06882233	0.08728249	Up	enoyl-[acyl-carrier-protein]
SMU_RS06135	353.1459037	398.865632	1.12783818	0.17356009	0.19636912	0.23127391	Up	enoyl-(acyl-carrier-protein) reductase
SMU_RS07930	577.6531173	2970.97934	5.14403248	2.36289975	2.75E-137	8.93E-135	Up	ketoacyl-ACP synthase III
SMU_RS07905	7273.030543	7944.70497	1.09224428	0.12729555	0.22051204	0.25722003	Up	beta-ketoacyl-ACP synthase II

SMU_RS07915	2976.936351	4840.62534	1.62646826	0.70174267	1.22E-16	4.82E-16	Up	ACP S-malonyltransferase
SMU_RS07935	736.7678964	1609.38632	2.18175262	1.12548753	4.00E-16	1.54E-15	Up	MULTISPECIES: MarR family transcriptional regulator
SMU_RS07940	3112.303236	7284.23437	2.34089873	1.22706253	7.79E-56	1.95E-54	Up	enoyl-CoA hydratase

Supplementary Table S4. Bacterial strains and plasmids used in this study

Strain or plasmid	Relevant characteristic	Source or reference
**Strain**		
S. mutans UA159	Wild-type stain	Laboratory stock
MUT16-R68A	Arginine 68 replaced by alanine of FtsZ; Spec	This study
MUT17-R68L	Arginine 68 replaced by leucine of FtsZ; Spec	This study
MUT18-R68G	Arginine 68 replaced by glycine of FtsZ; Spec	This study
**Plasmids**		
pET28a-smFtsZ	*E.coli* BL21(DE3), Kana^R	This study
pET28a-smFtsZ-MUT16-R68A	*E.coli* BL21(DE3), Kana^R	This study
pET28a-smFtsZ-MUT17-R68L	*E.coli* BL21(DE3), Kana^R	This study
pET28a-smFtsZ-MUT18-R68G	*E.coli* BL21(DE3), Kana^R	This study
pET28a-smFtsZ-truncated 1/319	*E.coli* BL21(DE3), Kana^R	This study
pET28a-smFtsZ-MUT20-Y247A	*E.coli* BL21(DE3), Kana^R	This study
pET28a-smFtsZ-MUT19-Y247F	*E.coli* BL21(DE3), Kana^R	This study
pET28a-smFtsZ-MUT21-E277L	*E.coli* BL21(DE3), Kana^R	This study
pET28a-smFtsZ-MUT22-E277D	*E.coli* BL21(DE3), Kana^R	This study
pET28a-smFtsZ-MUT23-E277A	*E.coli* BL21(DE3), Kana^R	This study
pET28a-smFtsZ-MUT44-E277T	*E.coli* BL21(DE3), Kana^R	This study
pET28a-smFtsZ-MUT26-T272V	*E.coli* BL21(DE3), Kana^R	This study
pET28a-smFtsZ-MUT41-T272C	*E.coli* BL21(DE3), Kana^R	This study
pET28a-smFtsZ-MUT25-S280A	*E.coli* BL21(DE3), Kana^R	This study
pET28a-smFtsZ-MUT45-S280R	*E.coli* BL21(DE3), Kana^R	This study

pUC19-UP-smFtsZ-mNG-DN	*E.coli* Top10, Amb^R	This study
pUC19-UP-smFtsZ_MUT16-R68A-mNG-DN	*E.coli* Top10, Amb^R	This study
pUC19-UP-smFtsZ_MUT17-R68L-mNG-DN	*E.coli* Top10, Amb^R	This study
pUC19-UP-smFtsZ_MUT18-R68G-mNG-DN	*E.coli* Top10, Amb^R	This study

Supplementary Table S5. Oligonucleotide primers used in this study

Primers	Sequence	Function
FtsZ_Sm-NcoⅠ-F 1	catgCcATGGCATTTTCATTTGATGCAG	*S. mutans* FtsZ
FtsZ_Sm-Xho Ⅰ-R 3	ccgCTCGAGACGATTCTTAAAGAAAGGAGG	
220828 R68A-F16	ATTAACCgcaGGTCTTGGTGCAGGAGGCCAA	pET28a-smFtsZ-MUT16-R68A/pUC19-UP-smFtsZ_MUT16-R68A-mNG-DN
220828 R68A-R16	AAGACCtgcGGTTAATTTAGGTCCAAGTTGAATAACTGT	
220828 R68L-F17	ATTAACCctgGGTCTTGGTGCAGGAGGC	pET28a-smFtsZ-MUT17-R68L/pUC19-UP-smFtsZ_MUT17-R68L-mNG-DN
220828 R68L-R17	AAGACCcagGGTTAATTTAGGTCCAAGTTGAATAACTGT	
220828 R68G-F18	ATTAACCgguGGTCTTGGTGCAGGAGGC	pET28a-smFtsZ-MUT18-R68G/pUC19-UP-smFtsZ_MUT18-R68G-mNG-DN
220828 R68G-R18	AAGACCaccGGTTAATTTAGGTCCAAGTTGAATAACTGT	
FtsZ_Sm-NcoⅠ-F 1	catgCcATGGCATTTTCATTTGATGCAG	pET28a-smFtsZ-truncated 1/319
smFtsZ-trca319-Xho Ⅰ-Nhis-R2	ccgCTCGAGttaGTCTGGCCGAACACCAGT	
220828 Y247A-F20	GCGATCgcaTCACCACTTCTTGAGACGACA	pET28a-smFtsZ-MUT20-Y247A
220828 Y247A-R20	GTGGTGAtgcGATCGCCTTGCGAGC	
220828 Y247F-F19	GCGATCTtTTCACCACTTCTTGAGACGACAATTGA	pET28a-smFtsZ-MUT19-Y247F
220828 Y247F-R19	GTGGTGAAaAGATCGCCTTGCGAGCAGCCT	

220828 E277L-F21	GAAGCTctgGAGGCTTCTGAAATTGTTAATCAAGC	pET28a-smFtsZ-MUT21-E277L
220828 E277L-R21	AGCCTCcagAGCTTCTGTCAGCGTCATATC	
220828 E277D-F22	GAAGCTgatGAGGCTTCTGAAATTGTTAATCAAGCTGC	pET28a-smFtsZ-MUT22-E277D
220828 E277D-R22	AGCCTCatcAGCTTCTGTCAGCGTCATATC	
220828 E277A-F23	GAAGCTgcaGAGGCTTCTGAAATTGTTAATCAAGCT	pET28a-smFtsZ-MUT23-E277A
220828 E277A-R23	AGCCTCtgcAGCTTCTGTCAGCGTCAT	
231218 E277T-F44	GAAGCTaccGAGGCTTCTGAAATTGTTAATCAAGCTGC	pET28a-smFtsZ-MUT44-E277T
231218 E277T-R44	AGCCTCggtAGCTTCTGTCAGCGTCATATCAAG	
220828 T272V-F26	GATATGgttCTGACAGAAGCTGAAGAGGCTTCTGA	pET28a-smFtsZ-MUT26-T272V
220828 T272V-R26	TGTCAGaacCATATCAAGGCCGCCGGTAAC	
231218 T272C-F41	GATATGtgtCTGACAGAAGCTGAAGAGGCTTCTGA	pET28a-smFtsZ-MUT41-T272C
231218 T272C-R41	TGTCAGacaCATATCAAGGCCGCCGGTAAC	
221209 S280A-F25	GAAGCTGAAGAGGCTgcaGAAATTGTTAATCAAG	pET28a-smFtsZ-MUT25-S280A
220828 S280A-R25	AATTTCtgcAGCCTCTTCAGCTTCTGTCAGC	
240102 S280R-F45v2	GAGGCTcgtGAAATTGTTAATCAAGCTGCAGGTCATGG	pET28a-smFtsZ-MUT45-S280R
240102 S280R-R45v2	AATTTCacgAGCCTCTTCAGCTTCTGTCAGC	
230409 pUC19-REV-F3	GATGCTGAAGAGATTGTTGATTTGCTGATAGAGTCGACCTGCAGGCATG	20230409 Amplified pUC19-REV
230409 pUC19-REV-R3	AAGTGAAATCActtCACGTTCTGGTGTGATGCTCGAGGATCCCCGGGTACCGA	
230409 UP-smFtsZ-F1	TCGGTACCCGGGGATCCTCGAGCATCACACCAGAACGTGaagTGATTTCACTT	20230409 Amplified UP-smFtsZ

230327smFtsZUp-1R	ATGCTGCATCAAATGAAAATGCCATTTTAATTTTTCCTCACTTTAATTTT	
230327smFtsZ-3F	AAAATTAAAGTGAGGAAAAATTAAAATGGCATTTTCATTTGATGCAGCAT	20230409 Amplified smFtsZ-mNG
230327linker_mNG-4R	TTTATTTGCTTGTAAATCCATTAttacttgtacagctcgtccatgc	
230327mNG-smFtsZDn-2F	gacgagctgtacaagtaaTAATGGATTTACAAGCAAATAAAGAACACGTT	20230409 Amplified DN-smFtsZ
230409 DN-smFtsZ-R2	CATGCCTGCAGGTCGACTCTATCAGCAAATCAACAATCTCTTCAGCATC	
230327linker_mNG-4F	TGGAAACACCTCCTTTCTTTAAGAATCGTGCAgaaGCTGCaGCtaaGgaAGC	230403 Amplified smFtsZ-mNG
230327linker_mNG-4R	TTTATTTGCTTGTAAATCCATTAttacttgtacagctcgtccatgc	
